# Circulation of non-falciparum malaria species in West Africa: A literature review from 2000 to 2024

**DOI:** 10.5281/zenodo.17938329

**Published:** 2025-12-15

**Authors:** Mamane N. Garba, Lamine M. Moustapha, Mamadou A. Diallo, Khadim Diongue, Mame C. Seck, Mahamadou Doutchi, Mouhamadou Ndiaye, Maman L. Ibrahim, Daouda Ndiaye, Aida S. Badiane

**Affiliations:** 1 Centre International de Recherche et de Formation en Génomique Appliquée et de Surveillance Sanitaire, Faculté de Médecine, de Pharmacie et d’Odontologie, Université Cheikh Anta Diop de Dakar, Sénégal.; 2 Faculté des Sciences et Techniques, Université André Salifou de Zinder, Niger.; 3 Faculté des Sciences de la Santé, Université André Salifou de Zinder, Niger.; 4 Centre de Recherche Médicale et Sanitaire de Niamey, Niger.

## Abstract

**Background:**

While malaria elimination efforts in West African countries focus primarily on *Plasmodium falciparum*, non-falciparum malaria species could replace *P. falciparum* if their elimination is neglected. It is within this malaria control and elimination context that this article reviews the circulation of non-falciparum malaria species in the West African region.

**Methods:**

Non-falciparum related articles with a focus on the West African sub-region, published between 2000 and 2024, were selected using internet search engines Google Search, PubMed Central®, and the National Library of Medicine. The methodologies, including the study type, period, population, clinical symptoms, and diagnostic techniques used, plus the frequency of non-falciparum species, were reviewed.

**Results:**

A total of 31 articles about non-falciparum malaria were reviewed, including 2 (6.5%) each from Benin, Burkina Faso, and Ivory Coast, 4 (12.9%) each from Ghana and Nigeria, 5 (16.1%) from Mali, 3 (9.7%) from Niger, and 9 (29.0%) from Senegal. Of the 31 reviewed papers, *P. falciparum* was reported in 29 (93.5%), *P. malariae* in 25 (80.6%), *P. ovale* in 24 (77.4%), *P. vivax* in 11 (35.5%). Overall, 17 (54.8%) of the review papers used RDTs including 12 (70.6%) PfHRP2-based RDTs, and 5 (29.4%) of the malaria Ag P.f/Pan-based RDTs.

**Conclusion:**

This review describes and provides a better understanding of the circulation of non-falciparum malaria species in West Africa. In addition, it underscores the need to adapt malaria diagnostics by using more sensitive techniques, and to modify control strategies to investigate asymptomatic Plasmodium infections.

## Introduction

Malaria remains a major public health problem in several countries around the world, particularly in sub-Saharan Africa. In 2023, there were an estimated 263 million new malaria cases in 83 countries worldwide, up from 252 million in 2022 and 226 million in 2015. The global tally of malaria deaths reached 597 000 in 2023 compared to 578 000 in 2015. The African region bears the heaviest burden of malaria, accounting for an estimated 94% of global cases and 95% of malaria-related deaths in 2023 [1].

Following many years of efforts made by the World Health Organization (WHO) to reduce malaria (providing technical support, recommendations and guidelines for malaria diagnosis, treatment, and prevention to countries), and by the affected countries (conducting surveillance and bednet distributions through National Malaria Control Programmes (NMCPs), developing research centres, and collaborating with various partners), hope of its elimination has begun to rise in some endemic countries [1]. Countries such as Mali, Niger, and Senegal continue to focus their diagnostic, treatment, prevention and surveillance efforts on *Plasmodium falciparum* while several studies have confirmed the increasing circulation of non-falciparum malaria species in these countries [2–4]. This could threaten progress toward malaria control and elimination.

In sub-Saharan Africa, human malaria is caused by *P. falciparum*, *P. malariae*, *P. ovale*, and *P. vivax*. More than 90% of reported malaria cases in Africa are caused by *P. falciparum* and, consequently, most detection methods focus on this species. However, in recent years, the reporting of non-falciparum cases has been increasing whilst *P. falciparum* has decreased in endemic regions where these malaria parasite species co-exist [3,5,6].

In endemic areas, Rapid Diagnostic Tests (RDTs) and microscopy (examination of Giemsastained malaria slides) are widely used for malaria diagnosis. Molecular assays such as the polymerase chain reaction (amplification of malaria parasite DNA, commonly known as PCR), and serological (detection of malaria parasite antigen) surveillance tools have proven to be more efficient in the era of malaria control and elimination. Several RDTs such as those based on the detection of the *P. falciparum* histidine-rich protein 2 (PfHRP2) and *P. falciparum* lactate dehydrogenase (PfLDH) antigens are used for *P. falciparum*. RDTs based on the detection of aldolase such as *P. vivax* aldolase (PvALDO), and RDTs based on the detection of lactate dehydrogenase, such as *Plasmodium* lactate dehydrogenase (pLDH) and *P. vivax* lactate dehydrogenase (PvLDH) are used for other non-falciparum malaria species. However, due to the primary role occupied by *P. falciparum* in malaria mortality and morbidity worldwide, strategies for malaria control in sub-Saharan Africa countries are mainly based on the use of PfHRP2 RDTs. Screening for non-falciparum malaria species is mostly performed in laboratories using molecular techniques [3,7,8]. Currently, few genomic studies of non-falciparum malaria species are conducted in West Africa [3,9]. Ghana is the leading country which published the complete sequencing data for *P. ovale curtisi*, and *P. ovale wallikeri* [10]; whereas some of non-falciparum genes were analysed by molecular tools to confirm their circulation in some sub-Saharan Africa countries [4,11,12].

Several recent studies conducted in sub-Saharan African regions reported increases of non-falciparum species and their coexistence with *P. falciparum*. The most worrying species is *P. vivax*, which was considered to only infect Duffy-positive populations has now been reported from Duffy-negative populations in various sub-Saharan countries [3,4,9,13]. Indeed, *P. vivax*, which was previously considered a benign parasite, may present similar symptoms to those of other types of malaria including cyclical fevers with chills, headache, weakness, vomiting, and diarrhoea. It has also been implicated in other more severe manifestations like acute renal failure, disseminated intravascular coagulation, acute respiratory distress syndrome complicating infection, hypoglycemia, and coma [14].

Since malaria elimination efforts in the West African sub-region are primarily focused on *P. falciparum*, non-falciparum species could replace *P. falciparum* should their elimination be neglected. It is within this malaria control and elimination context that this study aimed to review the circulation of non-falciparum malaria parasite species in West Africa between 2000 and 2024.

## Methodology

### Type of review

We conducted a narrative literature review of non-falciparum human malaria species in West Africa, using a structured search and selection process and a descriptive, narrative synthesis. No formal meta-analysis was planned.

### Eligibility criteria

We considered studies eligible if they met all of the following criteria: (i) conducted in at least one country of the West African sub-region, (ii) reported original data on the occurrence, prevalence, or circulation of at least one non-falciparum *Plasmodium* species (*P. malariae*, *P. ovale* spp., or *P. vivax*), either as mono-infection or in co-infection with *P. falciparum*; and (iii) used at least one parasitological diagnostic method (light microscopy of blood smears, rapid diagnostic tests [RDTs], molecular assays, genomic approaches, or serology). We included observational epidemiological designs (cross-sectional surveys, longitudinal cohorts, passive or active surveillance studies), national or subnational surveillance reports, and case reports or series with confirmed non-falciparum infections. We excluded narrative reviews, editorials, conference abstracts without full text, animal studies, and studies that did not provide species-specific data for non-falciparum malaria. No language restriction was applied at the search stage; however, all included articles were written in English or French.

### Information sources and search strategy

We searched Google Search (https://www.google.com), PubMed Central® (https://www.ncbi.nlm.nih.gov/pmc/), and the National Library of Medicine database (PubMed; https://www.ncbi.nlm.nih.gov) for articles published between 1 January 2000 and 31 December 2024. The year 2000 was chosen because it corresponds to the period when RDTs and molecular diagnostic tools began to be widely implemented in sub-Saharan Africa and when increasing numbers of *P. vivax* infections in Duffy-negative populations started to be reported. The upper limit (31 December 2024) corresponds to the last complete calendar year before this review was conducted.

Search terms combined species-level keywords with country names. An example of a Boolean search equation used in PubMed was:

(“*Plasmodium vivax*” OR “*P. vivax*” OR “*Plasmodium malariae*” OR “*P. malariae*” OR “*Plasmodium ovale*” OR “*P. ovale*” OR “non-falciparum”) AND (“Benin” OR “Burkina Faso” OR “Cape Verde” OR “Côte d’Ivoire” OR “Gambia” OR “Ghana” OR “Guinea” OR “Guinea-Bissau” OR “Liberia” OR “Mali” OR “Mauritania” OR “Niger” OR “Nigeria” OR “Senegal” OR “Sierra Leone” OR “Togo”).

Equivalent combinations of terms were used in the other databases. The search was restricted to the period 2000–2024 by applying publication-year filters. The last search was performed on 31 December 2024.

### Study selection

All records identified through the searches were imported into a reference manager and duplicates were removed. Two reviewers (MNG and MML) independently screened the titles and abstracts to assess potential eligibility. Full texts of potentially relevant articles were then reviewed in detail to determine final inclusion. Discrepancies between reviewers were resolved through discussion, and when necessary, consultation with a third reviewer (MAD). In total, 31 articles met all eligibility criteria and were included in the review.

### Data extraction and items

For each included study, we extracted the following information using a standardised form: country; first author and year; study period and design; target population (age group, symptomatic vs. asymptomatic); clinical features; diagnostic methods used (RDT type and target antigen, light microscopy, molecular or genomic techniques, serology); Duffy blood group status if reported; and the frequency of each *Plasmodium* species (including co-infections). These data are summarised in Tables 1–3.

### Quality assessment and risk of bias

Given the diversity of study designs and outcomes, we did not exclude studies based on a formal quality score. Instead, we conducted a qualitative assessment of potential sources of bias for each study, considering the sampling strategy (population-based vs. facility-based), inclusion criteria, completeness of reporting, and diagnostic methods used (including the sensitivity and specificity of the assays). These aspects were taken into account when interpreting the results and drawing conclusions, particularly when comparing findings across countries and diagnostic methods.

### Data synthesis and analysis

We employed a descriptive, narrative synthesis. Quantitative data were summarised using simple counts and percentages and organised by country, study design, target population, and diagnostic method. Frequencies of *P. falciparum* and non-falciparum species were tabulated (Tables 1–3), and we qualitatively compared methodological approaches and patterns of species occurrence across the West African sub-region. No statistical meta-analysis was attempted due to the heterogeneity in study designs, populations, and diagnostic tools.

## Results

A total of 31 non-falciparum malaria articles were reviewed including 2 (6.5%) each from Benin, Burkina Faso, and Ivory Coast, 4 (12.9%) each from Ghana and Nigeria, 5 (16.1%) from Mali, 3 (9.7%) from Niger, and 9 (29.0%) from Senegal (Table 1).

**Table 1:** Number of articles, and malaria species frequencies published per country (n = 31).

Country	Benin	Burkina Faso	Ivory Coast	Ghana	Mali	Niger	Nigeria	Senegal	Total
Number of articles (%)	2 (6.5)	2 (6.5)	2 (6.5)	4 (12.9)	5 (16.1)	3 (9.7)	4 (12.9)	9 (29.0)	31 (100)
P. falciparum	2 (6.9)	2 (6.9)	2 (6.9)	4 (13.8)	5 (17.2)	3 (10.3)	4 (13.8)	7 (24.1)	29 (100)
P. malariae	2(8)	2(8)	1(4)	4(16)	4(16)	3(12)	4(16)	5(20)	25 (100)
P. vivax	1 (9.9)	0	0	0	2 (18.2)	1 (9.9)	2 (18.2)	5 (45.5)	11 (100)
P. ovale	2 (8.3)	1 (4.2)	2 (8.3)	4 (16.7)	2 (8.3)	3 (12.5)	4 (16.7)	6(25)	24 (100)
P. ovale curtisi	0	0	0	1 (33.3)	1 (33.3)	0	0	1 (33.3)	3 (100)
P. ovale wallikeri	0	0	0	1(25)	1(25)	0	0	2(50)	4 (100)

### Study types, and clinical features of study participants

Among the reviewed articles, the types of studies included 2 (6.5%) each for case studies, passive case detection, prospective studies, and national surveillance; 4 (12.9%) for longitudinal studies, 14 (45.2%) for cross-sectional studies, and 5 (16.1%) for retrospective studies. Asymptomatic malaria populations harbouring non-falciparum species were reported in 15 (48.4%) studies. Only one (3.2%) study was conducted among Duffy-positive populations, the rest among Duffy-negative populations. All ages were affected by non-falciparum malaria species (Table 2).

**Table 2:** Characteristics of the articles reviewed per country.

Country	Ref.	Study period / design	Target population	Clinical profile	Diagnostic methods	Plasmo dium species	Key findings	Duffy status (if reported)
Benin	[27]	Longitudinal epidemiological surveillance (2009-2010)	Healthy blood donors	Asymptomatic populations	Light microscopy Elisa serology test Nested-PCR	*Pf* *Pm* *Pv* *Po*	**Microscopy:** 0. **Elisa** (n = 1234): r*Pv*MSP = 354 (28.7), r*Pv*CSPl = 266 (21.6), r*Pv*MSP + r*Pv*CSPl = 187 (15.2). PCR (n = 84): *Pf*= 20 (23); *Pm* = 2 (2.4); *Pv* = 13 (15.5); *Po* = 1 (1.2); *Pf*/*Pv* = 9 (10.7); *Pf*/*Pm*/*Pv* = 1 (1.2)	Duffy-negative popuiations
	[17]	Longitudinal epidemiological surveillance (2009-2010)	Healthy blood donors	Asymptomatic populations	- Elisa serology test	*Pf* *Pm* *Po*	**Elisa** (n = 1235): rP/MSPl + rP/AMAl= 1166 (94.4), r*Po*MSPl = 702 (56.8), r*Pm*MSPl = 834 (67.5), rP/MSPl + rP/AMAl + r*Po*MSPl + r*Pm*MSPl = 560 (45.3)	
Burkina Faso	[28]	Longitudinal epidemiological surveillance (2007-2010)	Children 3-15 yrs	Pain, fever history & patients with suspected malaria symptoms	- Light microscopy	*Pf* *Pm* *Po*	**Microscopy** (n = 830): *Pf* = 566 (68.2); *Pm* = 54 (6.5); *Po* = 9 (1.1); *Pf*/*Pm* = 36 (4.3); *Pf*/*Po* = 6 (0.7)	Duffy-negative populations
	[29]	Cross-sectional epidemiological surveillance (2017)	Children and adults aged 2 to >30 years	Fever history & malaria symptoms (= headache, chills, myalgia, dizziness, nausea, diarrhea)	RDT (SD BIOLINE Malaria Ag P.f/Pan) Light microscopy	*Pf* *Pm*	**RDT**: Not reported. **Microscopy** (n = 1199): P/= 393 (32.8); *Pm* = 9 (0.8); *Pf*/*Pm* = 20 (1.7)	
Ivory Coast	[30]	Longitudinal epidemiological surveillance (1993-2012)	French military personnel, regardless of location (France, French and foreign overseas territories)	Clinical and biological features (patients with positive blood smears)	RDTs (BinaxNOW Malaria TestPan/*Pv*/*Pf*) Light microscopy Quantitative Buffy Coat (QBC) Single-PCR Targeted sequencing	*Pf* *Po* *Po*c *Po*w	**RDT** (n = 315): Pan/*Pv*/*Pf*= 10. **Microscopy** (n = 315): *Po* = 305 (96.8). **QBC** (n = 78): *Po* = 67 (85.9). **PCR** (n = 3402): *Po*= 328 (9.6); *Pf*/*Po* = 27 (0.8). **Sequencing** (n = 19): *Po*c = 18 (94.7); *Po*w = 1 (5.3)	Duffy-positive populations
	[15]	Cross-sectional epidemiological surveillance (2015 & 2016)	Children aged 0 to 16 years	Asymptomatic populations and patients with fever, headache, abdominal pain	- Nested-PCR	*Pf* *Pm* *Po*	**PCR** (n = 360): P/= 201 (55.8); *Pm* = 50 (13.9); *Pm* = 16 (4.4); *Pf*/*Pm* = 39 (10.8); *Pf*/*Pm*/*Po* = 6 (1.7); *Pf*/*Po* = 9(2.5); *Pm*/*Po* = 1 (0.3)	Duffy-negative populations
Ghana	[10]	Cross-sectional epidemiological surveillance (2010)	Schoolchildren aged 5 to 17 years	Asymptomatic populations	RDTs (Malaria Pan Ag Shenyang LTH) Light microscopy Nested-PCR	*Pf* *Pm* *Po* *Po*c *Po*w	**RDT**: Not reported. **Microscopy** (n = 274): P/= 181 (66); *Pf*/*Pm* = 24 (8.8); *Pf*/*Po* = 3 (1.1). **PCR** (n = 270): P/= 115 (42.6); *Pm* = 76 (28.1); *Po* = 44 (16.3); *Po*c = 27 (10); *Po*w = 7 (2.6); *Po*c/*Po*w = 2 (0.7)	Duffy-negafive populations
	[31]	Cross-sectional epidemiological surveillance (2017)	All ages	Asymptomatic populations	RDTs (SD-BIOLINE Malaria Ag*Pf*HRP2) Light microscopy Nested-PCR	*Pf* *Pm* *Po*	**RDT** (n = 729): P/= 241 (33.1). **Microscopy** (n = 729): P/= 190 (26.1); *Pm* = 41 (5.6); *Pf*/*Pm* = 2 (0.3). **PCR** (n = 729): *Pf*= 401 (55); *Pm* = 9 (1.3); *Po* = 7 (0.9); *Pf*/*Pm* = 36 (4.9); *Pf*/*Po* = 25 (3.4); *Pf*/*Pm*/*Po* = 3 (0.4)	
	[7]	Cross-sectional epidemiological surveillance (2018)	Adults	Asymptomatic populations	RDTs (SD-BIOUNE Malaria Ag*Pf*HRP2) RDTs (BinaxNOW Malaria Test Pan/*Pv*/*Pf*) Nested-PCR	*Pf* *Pm* *Po*	**RDT**: Not reported. **PCR** (n=400): *Pf*= 266 (66.5); *Pm* = 33 (8.3); *Po* = 34 (8.5); *Po*c = 12 (3); *Po*w = 15 (3.8); *Pf*/*Pm* = 22 (5.5); *Pf*/*Po* = 20 (5); P//*Pm*/*Po* = 6 (1.5)	
	[16]	Cross-sectional epidemiological surveillance (2018)	Children and adults aged 1 to 94 years	Patients with suspected malaria symptoms	Light microscopy Nested-PCR	*Pf* *Pm* *Po*	**Microscopy** (n = 4937): *Pf* = 2284 (46.3); *Pm* = 1 (0.04); *Po* = 1 (0.04) Missing data = 323 (6.1). **PCR** (n =5260): P/= 3942 (74.9); *Pm* = 76 (1.4); *Po* = 46 (0.9); *Pf*/*Pm* = 64 (1.2); *Pf*/*Po* = 42 (0.9); *Pm*/*Po* = 1 (0.01); P//*Pm*/*Po* = 2(0.03)	
Mali	[32]	Retrospective epidemiological surveillance (2004- 2008)	All ages	Patient with suspected malaria symptoms	- Light microscopy	*Pf* *Pm*	**Microscopy** (n = 1623): *Pf* = 1606 (99); *Pm* = 17 (1)	Duffy-Negative populations
	[33]	National Malaria Control Programme surveillance (2012)	All ages.	Fever history & patients with suspected malaria symptoms	Light microscopy Nested-PCR	*Pf* *Pm* *Pv*	**Microscopy** (n = 88): *Po* = 25 (28.4) **PCR** (n =25): *Pf*/*Pv* = 11 (44); *Pm*/*Pv* = 4 (16)	
	[34]	Prospective cohort study (2009-2011)	Children aged < 1 to 6 years	Asymptomatic populations	Light microscopy Nested-PCR	*Pf* *Pv*	**Microscopy:** Not reported. **PCR** (n = 300): P/= 109 (36.3); Pd = 25 (8.3); *Pf*/*Pv* = 1 (0.3)	
	[2]	Cross-sectional epidemiological surveillance (2019)	Children older than 1 year and non-pregnant adults	Uncomplicated or asymptomatic populations	Light microscopy Nested-PCR	*Pf* *Pm* *Po* *Po*c *Po*w	**Microscopy:** Not reported. **PCR** (n = 4170): P/= 1497 (51.4); *Pm* = 73 (1.8); *Po*w = 3 (0.1); *Pf*/ *Pm* = 11 (0.3); *Pf*/*Po*w = 1 (0.02); *Pf*/*Po*c = 1 (0.02); *Pf*/*Po*c/*Pm* = 1 (0.02)	
	[23]	Retrospective epi- demioiogical data from 2016 to 2021.	Patients visiting the health center clinic, and community level: Passive and active screening cases (volunteers aged six months and more)	Malaria symptomatic and asymptomatic populations	- Light microscopy	*Pf* *Pm* *Po*	**Microscopy** (n = 8598): Passive (n = 6858): P/= 4069 (59.3); *Pm* = 103 (1.5); *Po* = 22 (0.3); *Pf*/*Pm* = 9 (0.1); *Pf*/*Po* = 2 (0.02); Active (n = 1740): P/= 336(19.3); *Pm* = 20 (1.2); *Po* = 1 (0.1); *Pf*/*Pm* = 2 (0.1); *Pm*/*Po* = 1 (0.1)	
Niger	[35]	Prospective, health center-based, clinical diagnostic evaluation (2017)	Children aged 3 to 59 months	Fever history & patients with suspected malaria symptoms	RDTs (SD-BIOLINE Malaria Ag*Pf*HRP2) Light microscopy	*Pf* *Pm* *Po*	**RDT** (n = 1946): P/= 585 (30.1). **Microscopy** (n = 1946): *Pf* = 401 (20.6); *Pm* = 4 (0.2); *Po* = 2 (0.1); *Pf*/*Po* = 3(0.2)	Duffy-negative populations
	[36]	Cross-sectional household-based malaria prevalence surveys(2018)	Children aged 3 months to 210 years	Malaria asymptomatic random points and household populations	- Light microscopy	*Pf* *Pm* *Po*	**Microscopy** (n = 1461): *Pf* = 574 (39.2); *Pm* = 3 (0.2); *Po* = 7 (0.5); *Pf*/*Po* = 11 (0.8) *Pf*/*Pm* = 4(0.3)	
	[4]	Cross-sectional Epidemiological surveillance (2022)	All ages.	Fever history & patients with suspected malaria symptoms	RDTs (SD-BIOLINE Malaria Ag*Pf*HRP2) Light microscopy PET-PCR	*Pf* *Pm* *Pv* *Po*	**RDT** (n = 340): P/= 130 (38.2). **Microscopy:** P/= 340 (100). **PCR** (n = 340): P/= 129 (37.9), *Pm* = 18 (5.3); *Pv* = 6 (1.8); *Po* = 1 (0.3); *Pf*/*Pm* = 71 (20.9); *Pf*/*Pv* = 15 (4.4); *Pf*/*Po* = 2 (0.6); *Pf*/*Pm*/*Pv* = 5 (1.5); *Pf*/*Pm*/*Po* = 1 (0.3); *Pm*/*Pv* = 1 (0.3)	
Nigeria	[25]	Cross-sectional Epidemiological surveillance (2016 & 2017)	Children aged 6 to 59 months	Fever history & patients with suspected malaria symptoms	RDT (Care Start® Malaria Ag *Pf*HRP2 Access Bio Inc) Light microscopy Nested-PCR Targeted sequencing	*Pf* *Pm* *Pv* *Po*	**RDT** (n = 436): P/= 300 (68.8). **Microscopy** (n = 436): P/= 135 (31.6) **PCR** (n = 436): P/= 219 (50.2); *Pv* = 1 (0.2); *Pf*/*Pm* = 12 (2.8); *Pf*/*Pv* = 4 (0.9); *Pf*/*Po* = 3 (0.6); *Pf*/*Pm*/*Pv* = 1 (0.2). Sequencing (n = 5): *Pv* = 5 (100)	Duffy-negative populations
	[9]	Cross-sectional Epidemiological surveillance (2016 & 2017)	Patients a 2 years	Patients with suspected malaria symptoms	RDT (Care Start® Malaria Ag *Pf*HRP2 Access Bio Inc) Light microscopy Nested-PCR Targeted sequencing	*Pf* *Pm* *Pv* *Po*	**RDT** (n = 242): P/= 187 (77.3) **Microscopy:** *Pf* =53 (21.9). **PCR** (n = 242): P/= 133 (55); *Pm* = 1 (0.4); *Po* = 1 (0.4); *Pv* = 3 (1.2); *Pf*/*Po* = 6 (2.5); *Pf*/*Pv* = 1 (0.4). Sequencing (n = 4): *Pv* = 4 (100)	
	[37]	Cross-sectional Epidemiological School surveillance (2016)	Adolescents aged 10 to 19 years	Asymptomatic populations	RDT (Care Start® Malaria Ag *Pf*HRP2 Access Bio Inc) Light microscopy Nested-PCR	*Pf* *Pm* *Po*	**RDT** (n = 658): P/= 310 (47.1) **Microscopy** (n = 658): *Pf* =60 (9.1) **PCR** (n = 658): P/= 158 (24); *Pm* = 66 (10); *Po* = 27 (4.1); *Pf*/*Pm* = 231 (35.1); *Pf*/*Po* = 39 (6); *Pf*/*Pm*/*Po* = 139 (21.1)	
	[5]	Nationwide HIV survey and assayed for Plasmodium anti- genemia (2018)	Children aged 0 to 14 years	Asymptomatic populations	IgG Anti-MSPl-19kD Assay RDT (Care Start® Malaria Ag *Pf*HRP2 Access Bio Inc) PET-PCR	*Pf* *Pm* *Po*	**IgG Serology** (n = 31234): *Pm* (anti-*Pm*MSPl IgG) = 10683 (34.2); *Po* (anti-*Po*MSPl IgG) = 3779 (12.1); *Pv* (anti-PoMSPl IgG) = 1968 (6.3). **RDT** (n = 31234): P/= 11963 (38.3). **PCR** (n = 1204): P/= 828 (68.7); *Pm* = 13 (1.1); *Po* = 4 (0.3); *Pf*/*Pm* = 149 (12.4); *Pf*/*Po* = 30 (2.5); *Pm*/*Po* = 2 (0.2); *Pf*/*Pm*/*Po* = 6 (0.5)	
Senegal	[38]	Case report (2014)	Patient aged 30- year-old man	Fever, headaches, myalgia, vomiting, diarrhoea, chills, asthenia, anorexia, anaemia, ascites, insomnia	RDTs (SD-BIOLINE Malaria Ag*Pf*HRP2) Light microscopy Nested-PCR	*Pm*	Identified by **Microscopy** and **PCR**	Duffy-Negative populations
	[39]	Retrospective serological surveillance (2009-2013)	Patients aged one to 65 years	Acute febrile illness	Nested-PCR Targeted sequencing	*Pf* *Pv* *Po*	**PCR** (n = 263): P/= 141 (53.6); *Pv* = 2 (0.8); *Pf*/*Po* = 19 (7.2); *Pf*/*Pv* = 2 (0.8). Sequencing (n = 4): *Pv* = 4 (100)	
	[21]	Case report (2016)	Patient aged 34- year-old man	Fever, vomiting	RDTs (SD-BIOLINE Malaria Ag*Pf*HRP2) Light microscopy Real time-PCR	*Po*	Identified by Microscopy and PCR	
	[11]	Passive case detection (2017)	Patients aged 6 months or older	Fever history & patients with suspected malaria symptoms	RDT (First Response Malaria Ag [HRP2]) Nested PCR High-resolution melting (HRM)	*Pf* *Pm* *Po* *Po*c Paw	**RDT** (n = 475): P/= 187 (39.4). **PCR** (n = 475): P/= 211 (44.4); *Pm* = 6 (1.3); *Po* = 6 (1.3). **HRM** (n = 4): *Po*c = 3 (75); *Po*re = 3 (75)	
	[40]	Retrospective serological surveillance (2010 to 2011)	Children aged 9 to 11 years	Asymptomatic populations	- Elisa serology test Nested-PCR	*Pf* *Pv*	**Elisa** (n = 188): *Pv* (Ag MSP1) = 99 (53); *Pf* (MSPlpl9) = 114 (61). **PCR** (n = 7): *Pv* = 5 (71.4)	
	[12]	Retrospective molecular surveillance (2010 to 2011)	Children aged 8 to 11 years	Asymptomatic populations	- Nested-PCR	*Pf* *Pv*	**PCR** (n = 192): P/= 59 (30.7); *Pv* = 15 (7.8)	
	[22]	Cross-sectional Epidemiological surveillance (2019)	Children and adults aged 6 months or older	Asymptomatic populations	RDT (Care Start® Malaria Ag *Pf*/Pan, Access Bio Inc) PET-PCR	*Pf* *Pm* *Po*	**RDT** (n = 122): P/= 49 (40.2); *Pf*/*Po* = 2 (1.6). **PCR** (n = 121): P/= 77 (63.6); *Pm* = 1 (0.8); *Po* = 1 (0.8); *Pf*/*Pm* = 1 (0.8); *Pf*/*Po* = 5 (4.1)	
	[24]	Cross-sectional Epidemiological surveillance (2015- 2021)	Patients at health facilities (6 months or older)	Malaria symptomatic populations with confirmed malaria	- PCR	*Pf* *Pm* *Pv* *Po*	**PCR** (n = 585): Uncomplicated malaria group (UM) (n = 353): P/= 115 (32.6); P//*Po*= 187 (53.1); *Pf*/*Pv*= 22 (6.2). Severe malaria group (SM) (n = 232): P/= 80 (34.5); P//*Po*= 99 (43.0); *Pf*/*Pv*= 20 (8.6).UM (n = 259): *Pm* = 4 (1.5); *Pv* = 40 (15.4); *Po* = 232 (89.6). SM (n = 163): *Pm* = 6 (3.7); *Pv* = 44 (27); *Po* = 138 (84.7). Death (n = 15): P//*Po*= 7 (46.7); *Pf*/*Pv* = 2 (13.3)	
	[3]	Passive case detection (2021)	Children and adults aged 6 months or older	Malaria asymptomatic & symptomatic populations	RDTs (SD-BIOLINE Malaria Ag*Pf*HRP2) PET-PCR Targeted amplicon sequencing	*Pf* *Pm* *Pv* *Po* Paw	**RDT** (n = 3000): P/= 96 (3.2). **PCR**: Not reported. **Sequencing** (n = 115): P/= 88 (76.5); *Pm* = 4 (3.5); *Pv* = 2 (1.7); *Po*w = 1 (0.8); *Pf*/*Pm* = 6 (5.2); *Pf*/*Pv* = 13 (11.3); *Pm*/*Pv* = 1 (0.8)	

### Diagnostic methods used

*Rapid Diagnostic Tests (RDTs)*: Among the 31 reviewed papers, different types of RDTs were used including 1 (3.2%) SD BIOLINE Malaria Ag P.f/Pan, 1 (3.2%) Malaria Pan Ag Shenyang LTH, 7 (22.6%) SD-BIOLINE Malaria Ag PfHRP2, 4 (12.9%) Care Start® Malaria Ag PfHRP2 Access Bio Inc, 1 (3.2%) Care Start® Malaria Ag Pf/Pan, Access Bio Inc, 1 (3.2%) First Response Malaria Ag [HRP2], and 2 (6.5%) BinaxNOW Malaria Test Pan/Pv/Pf. Overall, 54.8% (17/31) of the reviewed papers used RDTs including 12 (70.6%) of PfHRP2 based RDTs, and 5 (29.4%) of the Malaria Ag P.f/Pan based RDTs (Tables 2 and 3).

**Table 3:** Frequency of the methodology characteristics of the reviewed papers (n = 31).

Study type	Clinical features & Diagnosis				
	RDTs		Light microscopy, PCR & Genomics	Duffy status	Serology
Frequency No. (%)						
Case report 2 (6.5)	SD BIOLINE Malaria Ag Pi/Pan 1 (3.2)		Light Microscopy 20 (64.5)	Duffy negative populations 30 (96.8)	Elisa serology antigen recombinant tests + IgG Anti- MSPl-19kD Assays 4 (12.9)
Passive case detection 2 (6.5)	Malaria Pan Ag Shenyang LTH 1(3.2) ‘		Single-PCR 2 (6.5)	Duffy positive populations 1 (3.2)	
Longitudinal 4 (12.9)	SD-BIOLINE Malaria Ag PIHRP2 7 (22.6)		Nested-PCR 17 (54.8)		
Cross-sectional 14 (45.2)	Care Start® Malaria Ag PfHRP2 Access Bio Inc 4 (12.9)		PET-PCR 3 (9.7)		
Retrospective 5(16.1)	Care Start® Malaria Ag Pf / Pan, Access Bio Inc 1 (3.2)		HRM 1 (3.2)		
Prospective 2 (6.5)	First Response Malaria Ag [HRP2] 1 (3.2)		Real time-PCR 1 (3.2)		
National Surveillance 2 (6.5)	BinaxNOW Malaria Test Pan/Pv/Pf 2 (6.5)		Targeted sequencing 7 (22.6)		
	Total RDTs 17 (54.8)				
	PfHRP2 based RDTs 12 (70.6)	Malaria Ag P.f/Pan based RDTs 5 (29.4)			

*Light microscopy*: The light microscopy tests were performed in 30 (96.8%) of the reviewed papers (Tables 2 and 3).

*Molecular assays*: PCR and genomic techniques including 2 (6.5%) single-PCR, 17 (54.8%) Nested-PCR, 3 (9.7%) PET-PCR, 1 (3.2%) HRM, 1 (3.2%) Real time-PCR, and 5 (16.1%) targeted sequencing were performed (Tables 2 and 3).

*Serology assays*: Seroprevalence tests were reported from 4 studies including two Elisa serology antigen recombinant tests and two multiplex bead-based IgG Anti-MSP1-19kD Assays were performed (Tables 2 and 3).

### Frequency of reported non-falciparum malaria per country

The reviewed articles report 29 (93.5%) of the reported cases of *P. falciparum* infection, including 2 (6.9%) for each Benin, Burkina Faso, and Côte d'Ivoire; 4 (13.8%) for each Ghana, and Nigeria; 5 (17.2%), 3 (10.3%), and 7 (22.2%) respectively for Mali, Niger, and Senegal. Also, 25 (80.6%) of the reported cases of *P. malariae* infection were noted, including 2 (8.0%) for Benin, and Burkina Faso; 1 (4.0%) for Côte d'Ivoire, 4 (16.0%) for Ghana, Mali, and Nigeria, 3 (12.0%) for Niger, and 5 (20.0%) for Senegal. This work also revealed 24 (77.4%) of *P. ovale* cases reported including 2 (8.3%) for Benin, Côte d'Ivoire, and Mali; 1 (4.2%) for Burkina Faso, 4 (16.7%) for Ghana and Nigeria; 3 (12.5%) and 6 (25.0%) respectively for Niger and Senegal. The *P. ovale* cases included subspecies, notably 3 (9.7%) of *P. ovale curtisi* (1 in Ghana, 1 in Mali, and 1 in Senegal) and 4 (12.9%) of *P. ovale wallikeri* (1 in Ghana, 1 in Mali, and 2 in Senegal). For *Plasmodium vivax*, 11 (35.5%) of cases were reported including 1 (9.9%) for Benin, 2 (18.2%) for Mali and Nigeria, 1 (9.9%) for Niger, and 5 (45.5%) for Senegal. It should be noted that only 3 (9.7%) of *P. vivax* cases were confirmed by sequencing. (Tables 1 and 2).

## Discussion

This literature review focused on studies conducted on the circulation of non-falciparum malaria species in West Africa that were published between 2000 and 2024. As *P. falciparum* malaria is decreasing in several endemic countries, other species are receiving increased attention due to the elimination goal, which should address symptomatic and asymptomatic infections due to all species. The objective of this review was to assess the burden of non-falciparum malaria species in the sub-Saharan Africa for control and elimination strategies.

This review highlighted non-falciparum malaria circulation in West Africa. Most studies were conducted among Duffy-negative populations, and almost a third confirmed the circulation *P. vivax* in the studied populations. Due to their well-known and well described virulence, non-falciparum malaria could become a burden to the West African sub-region [2,4,15,16]. However, NMCPs and their partners focus their funds and efforts on *P. falciparum* control and elimination. Consequently, the lack of intensive research on these non-falciparum malaria species could hinder malaria control efforts [17].

In the past decade, many sub-Saharan countries embraced the hope of making malaria elimination a reality. However, this hope has met several concerns that have been identified including submicroscopic diagnosis of malaria parasites, the RDT HRP2 gene deletion, and the increasing frequency of *P. vivax* in Duffy-negative populations. In addition, *P. vivax* may cause clinically significant malaria and has implications for treatment as hypnozoites are not cleared by artemisinin-based combination therapy and are implicated in relapses, which are estimated to account for 50% to 96% of all post-treatment *P. vivax* recurrences in Thailand and Ethiopia [18,19]. These obstacles have led the NMCPs and various partners to consider improving the technical diagnostic platforms to include molecular techniques and genomics in several countries of this region. In addition to *P. malariae*, *P. vivax*, and *P. ovale*, the two other sub-species of *P. ovale* (*P. ovale curtisi* and *P. ovale wallikeri*) were reported [10,20,21]. This could also present additional challenges for the diagnosis, treatment, and prevention of the three non-falciparum malaria species whose identification remains poorly mastered by microscopists in health centres and hospitals. Non-falciparum malaria species were present among asymptomatic malaria populations and were reported in about half of the reviewed papers 48.4% (15/31), confirming the role of non-falciparum malaria species in asymptomatic populations [22–24]. Therefore, active case detection in community surveillance is necessary to better map the circulation of non-falciparum species to diagnose asymptomatic subjects in the community for better interventions. These reports underline the need to conduct more active case detection in community surveillance to help detect non-falciparum malaria species, especially in Senegal, a country that is considered to be a potential malaria elimination candidate. Given the high proportion of these non-falciparum malaria species circulating in these countries, the introduction and scale up of pan-LDH–only RDTs and combined *P. falciparum* pLDH and pan-LDH RDTs should be considered [3,4,6]. Noting the rise of non-falciparum malaria species and their coexistence with the *P. falciparum* species in several malaria endemic areas, non-falciparum malaria species may become a burden in West Africa if their NMCPs delay acknowledging the urgency of their existence while designing malaria control and elimination strategies.

A review of the diagnostic tests reported indicates that Pan RDTs constituted approximately one-third of PfHRP2 RDTs. This finding suggests that the primary focus of the ongoing efforts to combat *P. falciparum* malaria is being adequately addressed, while the effort to combat *P. falciparum* and *P. vivax* malaria in these countries may be less effective. Molecular biology persists as a reliable instrument in the development of laboratory screening methodologies for malaria species, as evidenced in Table 2. In accordance with numerous other studies, this review further substantiates its ultrasensitivity when contrasted with RDTs and light microscopy for the expeditious identification of *P. falciparum* and non-falciparum malaria species within a laboratory setting [4,22,25].

The four seroprevalence results reported in this review support the use of serology as a tool for malaria surveillance in endemic areas. However, as reported in several studies, to better estimate changes in the force of infection over the long and short term in areas with low malaria endemicity, further information on the longevity of the antibody responses is necessary. Serology assays have been demonstrated to play a pivotal role in detecting recent malaria transmission, as they can identify recent exposure, thereby providing an indirect measure of hypnozoite carriage in individuals. This makes them a potentially powerful surveillance tool, especially in the context of malaria elimination. Furthermore, it has been demonstrated to be a reliable screening method for the simultaneous detection of antibodies against *Plasmodium* antigens [26].

This review highlights the current place of non-falciparum malaria in the current malaria control and elimination era, providing a better understanding of its circulation in the West African sub-region. It also underlines the importance of asymptomatic malaria in disease transmission, which should be considered by the region’s NMCPs as an additional target to achieve the malaria elimination goal. One limitation of this review is that we may have overlooked studies that were not identified during the search criteria outlined in the methodology section. Other limitations of this review include the relatively small number of studies conducted on the circulation of non-falciparum malaria species in West Africa during this time period, and the underestimation of the real rate of non-falciparum species circulating in the region. Indeed, for malaria diagnosis, most of the countries from the reviewed articles use PfHRP2 based RDTs which are specific to *P. falciparum* diagnosis, and the light microscopy which relies on the experience of microscopists. Effectively, due to the preponderance and the higher parasite densities of *P. falciparum* than the other species, microscopists will be less accustomed to the detection of non-falciparum species. Therefore, several non-falciparum species could escape RDTs and light microscopy [4]. It should be noted that molecular and serological surveillance techniques have proven to be more efficient and are ideal tools to be used by each country for malaria surveillance, but they are still not accessible to all due to their high cost.

These limitations imply that our estimates likely underestimate the true circulation of non-falciparum species in West Africa, particularly low-density and mixed-species infections, and that our conclusions should be interpreted with appropriate caution.

## Conclusions

This review offers a more nuanced understanding of the circulation of non-falciparum malaria species in West African countries, highlighting the potential diagnostic, therapeutic, and preventive challenges posed by their presence. The analysis revealed that most studies were conducted among asymptomatic populations, where all age groups were affected by non-falciparum malaria species. Consequently, this review underscores the necessity to adapt malaria diagnostic tools and therapeutic management, as well as to train microscopists for the recognition, detection, and identification of non-falciparum malaria species circulating in the West African region to advance the goals of malaria control and elimination. Nevertheless, the diagnostic gaps, limited number of studies from several countries, and probable under-detection of low-density and mixed infections highlight the need for more comprehensive, standardised surveillance of non-falciparum malaria across the West African sub-region.

## References

[1] World Health Organization: World Malaria Report 2024. https://tinyurl.com/3d35ec9a (Accessed 9 December 2025).

[2] Dembele L, Aniweh Y, Diallo N, Sogore F. et al.: *Plasmodium malariae* and *Plasmodium falciparum* comparative susceptibility to antimalarial drugs in Mali. J. Antimicrob. Chemother. 2021, 76: 2079–2087. Doi: 10.1093/jac/dkab133 34021751

[3] Badiane AS, Ngom B, Ndiaye T, Cunningham D. et al.: Evidence of *Plasmodium vivax* circulation in western and eastern regions of Senegal: implications for malaria control. Malar. J. 2024, 23:149. Doi: 10.1186/s12936-024-04932-z 38750583 PMC11097470

[4] Garba MN, M. Moustapha L, Sow D, Karimoun A. et al.: Circulation of non-falciparum species in Niger: Implications for malaria diagnosis. Open Forum Infect. Dis. 2024, 11: ofae474. Doi: 10.1093/ofid/ofae474 39282631 PMC11394099

[5] Herman C, Leonard CM, Uhomoibhi P, Maire M. et al.: Non-falciparum malaria infection and IgG seroprevalence among children under 15 years in Nigeria, 2018. Nat. Commun. 2023, 14: 1360. Doi: 10.1038/s41467-023-37010-0 36914649 PMC10011577

[6] Oboh MA, Oyebola KM, Idowu ET, Badiane AS. et al.:. Rising report of *Plasmodium vivax* in sub-Saharan Africa: Implications for malaria elimination agenda. Sci. Afr. 2020, 10: e00596. Doi: 10.1016/j.sciaf.2020.e00596

[7] Heinemann M, Phillips RO, Vinnemeier CD, Rolling CC. et al.: High prevalence of asymptomatic malaria infections in adults, Ashanti Region, Ghana, 2018. Malar. J. 2020, 19: 366. Doi: 10.1186/s12936-020-03441-z 33046056 PMC7552528

[8] Dao F, Dembele L, Diarra B, Sogore F. et al.: The prevalence of human *Plasmodium* species during peak transmission seasons from 2016 to 2021 in the rural commune of Ntjiba, Mali. Trop. Med. Infect. Dis. 2023, 8: 438. Doi: 10.3390/tropicalmed8090438 37755899 PMC10535850

[9] Oboh MA, Singh US, Ndiaye D, Badiane AS. et al.: Presence of additional *Plasmodium vivax* malaria in Duffy negative individuals from Southwestern Nigeria. Malar. J. 2020, 19: 229. Doi: 10.1186/s12936-020-03301-w 32590997 PMC7318376

[10] Dinko B, Oguike MC, Larbi JA, Bousema T. et al.: Persistent detection of *Plasmodium falciparum*, *P. malariae*, *P. ovale curtisi* and *P. ovale wallikeri* after ACT treatment of asymptomatic Ghanaian school-children. Int. J. Parasitol. Drugs Drug Resist. 2013, 3: 45–50. Doi: 10.1016/j.ijpddr.2013.01.001 24533292 PMC3862415

[11] Daniels RF, Deme AB, Gomis JF, Dieye B. et al.: Evidence of non-*Plasmodium falciparum* malaria infection in Kédougou, Sénégal. Malar. J. 2017, 16: 9. Doi: 10.1186/s12936-016-1661-3 28049489 PMC5209815

[12] Niang M, Sane R, Sow A, Sadio BD. et al.: Asymptomatic *Plasmodium vivid* infections among Duffy-negative population in Kedougou, Senegal. Trop. Med. Health. 2018, 46: 45. Doi: 10.1186/s41182-018-0128-3 30618490 PMC6311047

[13] Wilairatana P, Masangkay FR, Kotepui KU, De Jesus Milanez G. et al.: Prevalence and risk of *Plasmodium vivax* infection among Duffy-negative individuals: a systematic review and meta-analysis. Sci. Rep. 2022, 12: 3998. Doi: 10.1038/s41598-022-07711-5 35256675 PMC8901689

[14] Rahimi BA, Thakkinstian A, White NJ, Sirivichayakul C. et al.: Severe vivax malaria: a systematic review and meta-analysis of clinical studies since 1900. Malar. J. 2014, 13: 481. Doi: 10.1186/1475-2875-13-481 25486908 PMC4364574

[15] Miezan AJS, Gnagne AP, Bedia-Tanoh A V., Kone EGM. et al.: Molecular epidemiology of non-falciparum *Plasmodium* infections in three different areas of the Ivory Coast. Malar. J. 2023, 22: 211. Doi: 10.1186/s12936-023-04639-7 37468917 PMC10357878

[16] Amoah LE, Asare KK, Dickson D, Anang S. et al.: Nationwide molecular surveillance of three *Plasmodium* species harboured by symptomatic malaria patients living in Ghana. Parasit. Vectors 2022, 15: 40. Doi: 10.1186/s13071-022-05153-6 35090545 PMC8796507

[17] Doderer-Lang C, Atchade PS, Meckert L, Haar E. et al.: The ears of the African elephant: unexpected high seroprevalence of *Plasmodium ovale* and *Plasmodium malariae* in healthy populations in Western Africa. Malar. J. 2014, 13: 240. Doi: 10.1186/1475-2875-13-240 24946685 PMC4071337

[18] Demissie Y, Ketema T: Complicated malaria symptoms associated with *Plasmodium vivax* among patients visiting health facilities in Mendi town, Northwest Ethiopia. BMC Infect. Dis. 2016, 16: 436. Doi: 10.1186/s12879-016-1780-z 27549864 PMC4994234

[19] Chu CS, Stolbrink M, Stolady D, Saito M. et al.: Severe Falciparum and Vivax Malaria on the Thailand-Myanmar Border: A Review of 1503 Cases. Clin. Infect. Dis. 2023, 77: 721–728. Doi: 10.1093/cid/ciad262 37144342 PMC10495127

[20] Oguike MC, Betson M, Burke M, Nolder D. et al.: *Plasmodium ovale curtisi* and *Plasmodium ovale wallikeri* circulate simultaneously in African communities. Int. J. Parasitol. 2011, 41: 677–683. Doi: 10.1016/j.ijpara.2011.01.004 21315074 PMC3084460

[21] Diallo MA, Badiane AS, Diongue K, Deme A. et al.: Non-falciparum malaria in Dakar: a confirmed case of *Plasmodium ovale wallikeri* infection. Malar. J. 2016, 15: 429. Doi: 10.1186/s12936-016-1485-1 27557982 PMC4997729

[22] Badiane AS, Ndiaye T, Thiaw AB, Binta DA. et al.: High prevalence of asymptomatic *Plasmodium* infection in Bandafassi, South-East Senegal. Malar. J. 2021, 20: 218. Doi: 10.1186/s12936-021-03746-7 33980241 PMC8117620

[23] Dao F, Dembele L, Diarra B, Sogore F. et al.: The prevalence of human *Plasmodium* species during peak transmission seasons from 2016 to 2021 in the rural commune of Ntjiba, Mali. Trop. Med. Infect Dis. 2023, 8: 438. Doi: 10.3390/tropicalmed8090438 37755899 PMC10535850

[24] Diagne A, Sambe BS, Gaba FM, Sarr I. et al.: Variable effects of non-falciparum species infections on malaria disease severity in high transmission regions in Senegal. Trop. Med. Health 2024, 52: 93. Doi: 10.1186/s41182-024-00655-8 39633482 PMC11616377

[25] Oboh MA, Badiane AS, Ntadom G, Ndiaye YD. et al.: Molecular identification of *Plasmodium* species responsible for malaria reveals *Plasmodium vivax* isolates in Duffy negative individuals from southwestern Nigeria. Malar. J. 2018, 17: 439. Doi: 10.1186/s12936-018-2588-7 30486887 PMC6263541

[26] Plucinski M, Aidoo M, Rogier E: Laboratory detection of malaria antigens: a strong tool for malaria research, diagnosis, and epidemiology. Clin. Microbiol Rev. 2021, 34: e0025020. Doi: 10.1128/cmr.00250-20 34043447 PMC8262808

[27] Poirier P, Doderer-Lang C, Atchade PS, Lemoine J-P. et al.: The hide and seek of *Plasmodium vivax* in West Africa: report from a large-scale study in Beninese asymptomatic subjects. Malar. J. 2016, 15: 570. Doi: 10.1186/s12936-016-1620-z 27887647 PMC5123334

[28] Gnémé A, Guelbéogo WM, Riehle MM, Tiono AB. et al.: *Plasmodium* species occurrence, temporal distribution and interaction in a child-aged population in rural Burkina Faso. Malar. J. 2013, 12: 67. Doi: 10.1186/1475-2875-12-67 23421809 PMC3583752

[29] Yaro JB, Tiono AB, Ouedraogo A, Lambert B. et al.: Risk of *Plasmodium falciparum* infection in south-west Burkina Faso: potential impact of expanding eligibility for seasonal malaria chemoprevention. Sci. Rep. 2022, 12: 1402. Doi: 10.1038/s41598-022-05056-7 35082312 PMC8791962

[30] de Laval F, Simon F, Bogreau H, Rapp C. et al.: Emergence of *Plasmodium ovale* malaria among the French Armed Forces in the Republic of Ivory Coast: 20 years of clinical and biological experience. Clin. Infect. Dis. 2014, 58: e122–128. Doi: 10.1093/cid/ciu021 24429426

[31] Amoah LE, Donu D, Abuaku B, Ahorlu C. et al.: Probing the composition of *Plasmodium* species contained in malaria infections in the Eastern region of Ghana. BMC Public Health 2019, 19: 1617. Doi: 10.1186/s12889-019-7989-1 31791319 PMC6889690

[32] Ba O: Etude de la saisonnalité du paludisme à *Plasmodium falciparum* en milieu urbain de Bamako. Thèse de Doctorat. ADHL Mali Home Université des Sciences, des Techniques et des Technologies de Bamako Faculté de Médecine et d’Odontostomalogie. 2010. https://tinyurl.com/33cm7v4v (Accessed 10 December 2025).

[33] Bernabeu M, Gomez-Perez GP, Sissoko S, Niambélé MB. et al.: *Plasmodium vivax* malaria in Mali: a study from three different regions. Malar. J. 2012, 11: 405. Doi: 10.1186/1475-2875-11-405 23217064 PMC3547733

[34] Niangaly A, Karthigayan Gunalan, Amed Ouattara, Coulibaly D. et al.: *Plasmodium vivax* infections over 3 Years in Duffy Blood Group Negative Malians in Bandiagara, Mali. Am. J. Trop. Med. Hyg. 2017, 97: 744–752. Doi: 10.4269/ajtmh.17-0254 28749772 PMC5590610

[35] Coldiron ME, Assao B, Langendorf C, Sayinzoga-Makombe N. et al.: Clinical diagnostic evaluation of HRP2 and pLDH-based rapid diagnostic tests for malaria in an area receiving seasonal malaria chemoprevention in Niger. Malar. J. 2019, 18: 443. Doi: 10.1186/s12936-019-3079-1 31878947 PMC6933886

[36] Coldiron ME, Assao B, Guindo O, Sayinzoga-Makombe N. et al.: Prevalence of malaria in an area receiving seasonal malaria chemoprevention in Niger. Malar. J. 2021, 20: 419. Doi: 10.1186/s12936-021-03953-2 34689782 PMC8543849

[37] Abdulraheem MA, Ernest M, Ugwuanyi I, Abkallo HM. et al.: High prevalence of *Plasmodium malariae* and *Plasmodium ovale* in co-infections with *Plasmodium falciparum* in asymptomatic malaria parasite carriers in southwestern Nigeria. Int. J. Parasitol. 2022, 52 : 23–33. Doi: 10.1016/j.ijpara.2021.06.003 34390743

[38] Badiane AS, Diongue K, Diallo S, Ndongo AA. et al.: Acute kidney injury associated with *Plasmodium malariae* infection. Malar. J. 2014, 13: 226. Doi: 10.1186/1475-2875-13-226 24906879 PMC4062646

[39] Niang M, Thiam LG, Sow A, Loucoubar C. et al.: A molecular survey of acute febrile illnesses reveals *Plasmodium vivax* infections in Kedougou, southeastern Senegal. Malar. J. 2015, 14: 281. Doi: 10.1186/s12936-015-0808-y 26186936 PMC4506577

[40] Niang M, Diop F, Niang O, Sadio BD. et al.: Unexpected high circulation of *Plasmodium vivax* in asymptomatic children from Kédougou, southeastern Senegal. Malar. J. 2017, 16: 497. Doi: 10.1186/s12936-017-2146-8 29284488 PMC5747145

